# Antimicrobial Peptide Arenicin-1 Derivative Ar-1-(C/A) as Complement System Modulator

**DOI:** 10.3390/md18120631

**Published:** 2020-12-10

**Authors:** Ilia A. Krenev, Ekaterina S. Umnyakova, Igor E. Eliseev, Yaroslav A. Dubrovskii, Nikolay P. Gorbunov, Vladislav A. Pozolotin, Alexei S. Komlev, Pavel V. Panteleev, Sergey V. Balandin, Tatiana V. Ovchinnikova, Olga V. Shamova, Mikhail N. Berlov

**Affiliations:** 1 Department of General Pathology and Pathological Physiology, Institute of Experimental Medicine, Acad. Pavlov Str. 12, 197376 Saint Petersburg, Russia; il.krenevv13@yandex.ru (I.A.K.); niko_laygo@mail.ru (N.P.G.); vlad.yugra.nyagan@gmail.com (V.A.P.); witcher-lex@yandex.ru (A.S.K.); oshamova@yandex.ru (O.V.S.); berlov.mn@iemspb.ru (M.N.B.); 2 Faculty of Chemistry, Saint Petersburg State University, Universitetskaya Emb, 7/9, 199034 Saint Petersburg, Russia; dubrovskiy.ya@gmail.com; 3 Nanobiotechnology Laboratory, Alferov University, Khlopin Str. 8/3, 194021 Saint Petersburg, Russia; eliseev@spbau.ru; 4 Almazov National Medical Research Centre, Akkuratov Str, 2, 197341 Saint Petersburg, Russia; 5 M.M. Shemyakin and Yu. A. Ovchinnikov Institute of Bioorganic Chemistry, Russian Academy of Sciences, Miklukho-Maklaya Str., 16/10, 117997 Moscow, Russia; alarm14@gmail.com (P.V.P.); serb@ibch.ru (S.V.B.); ovch@ibch.ru (T.V.O.); 6 Department of Biotechnology, I.M. Sechenov First Moscow State Medical University, Trubetskaya Str., 8-2, 119991 Moscow, Russia

**Keywords:** antimicrobial peptide, arenicin, complement system, complement regulation

## Abstract

Antimicrobial peptides (AMPs) are not only cytotoxic towards host pathogens or cancer cells but also are able to act as immunomodulators. It was shown that some human and non-human AMPs can interact with complement proteins and thereby modulate complement activity. Thus, AMPs could be considered as the base for complement-targeted therapeutics development. Arenicins from the sea polychaete *Arenicola marina*, the classical example of peptides with a β-hairpin structure stabilized by a disulfide bond, were shown earlier to be among the most prospective regulators. Here, we investigate the link between arenicins’ structure and their antimicrobial, hemolytic and complement-modulating activities using the derivative Ar-1-(C/A) without a disulfide bond. Despite the absence of this bond, the peptide retains all important functional activities and also appears less hemolytic in comparison with the natural forms. These findings could help to investigate new complement drugs for regulation using arenicin derivatives.

## 1. Introduction

Antimicrobial peptides (AMPs) are short, predominantly cationic polypeptide molecules that possess toxic activity against different pathogens: bacteria, enveloped viruses, fungi, parasites, etc. These peptides were first discovered to be cytotoxic agents against bacteria but later more and more data appeared about the immunoregulatory and wound-healing activities of different AMPs. For example, human defensins possess chemotactic activities for some immune cells [1,2,3,4] and stimulate angiogenesis [5] and collagen synthesis in fibroblasts [6]. They also participate in autoimmune processes [7]. All these findings give evidence that AMPs could be used as a promising basis for the development of new generations of antibiotic and anticancer drugs [8,9,10]. These substances not only participate in host defense against pathogens but they also possess immunomodulatory activity [11]. In particular, human and non-human AMPs were found to bind complement proteins and to modulate human complement system activity [12,13,14,15,16,17,18].

The complement system is a network containing more than 30 soluble and membrane-associated proteins that consistently activate each other through limited proteolysis reactions. This leads to anaphylatoxin (C3a and C5a) formation, to opsonization of the target surfaces by derivatives of the complement proteins and to membrane attack complex (MAC) assembly, which could provoke the complement-mediated lysis of target cells that is mainly typical of Gram-negative bacteria [19]. The dysregulation of the complement can cause different dangerous inflammatory diseases. They can be directly associated with excessive activation of the complement system (age-related macular degeneration, atypical hemolytic uremic syndrome, type II membranoproliferative glomerulonephritis, paroxysmal nocturnal hemoglobinuria), as well as with insufficient complement activation [20]. One of the most severe problems of modern medicine is the absence of available therapeutic agents that regulate complement activation. Thus, we are searching for such substances among AMPs.

We showed earlier that one of the most promising candidates appeared to be arenicin-1 (Ar-1), the antimicrobial peptide from *Arenicola marina*, that is able to modulate complement activity [18]. Arenicins were discovered by Ovchinnikova and co-workers [21], and there are three isoforms in total, of which two, Ar-1 and Ar-2, differ in a single amino acid residue [22]. Our next aim was to modify the primary structure of this peptide to achieve the following goals: (1) to improve the ability to inhibit complement activity to treat diseases caused by hyperactivation of this system; (2) to lower cytotoxic activity towards the host cells decrease undesirable side effects; (3) to save the antimicrobial activity of the peptide for the host defense against bacteria while the complement is inhibited. Here, we made an Ar-1 derivative without the intramolecular disulfide bond, where Cys3 and Cys20 were replaced by Ala and thus we named this peptide Ar-1-(C/A). This design was developed to check whether this bond is critical for the structural stability and biological activity, i.e., antimicrobial activity against Gram-positive and Gram-negative bacteria, cytotoxic activity toward human erythrocytes and the ability to regulate the human complement system. The main goal of this investigation was to identify the differences in the action of natural arenicins Ar-1, Ar-2 and analog Ar-1-(C/A).

## 2. Results

**Figure 1:**
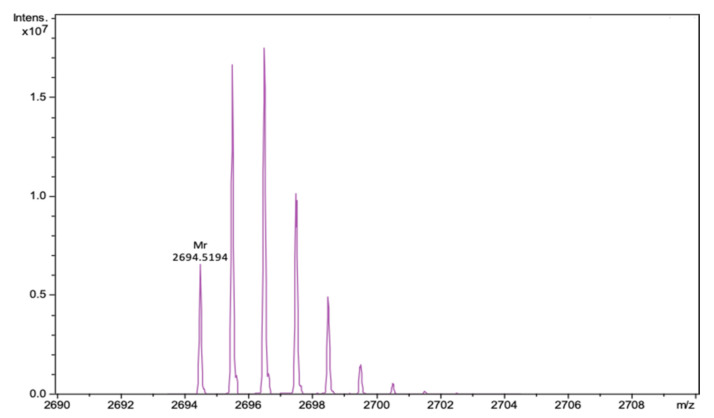
Fragment of the mass spectrum of Ar-1(C/A) after deconvolution for neutral molecule. The calculated monoisotopic mass of the peptide is 2694.525, the experimentally determined *m*/*z* value is 2694.519.

We performed successful peptide synthesis of a new derivative of natural Ar-1. Our variant contains two alanine residues (Ala^3^ and Ala^20^) instead of two cysteines, thus, it is not stabilized by a disulfide bond. The mass spectrum of Ar-1-(C/A) is presented in Figure 1.

**Figure 2:**
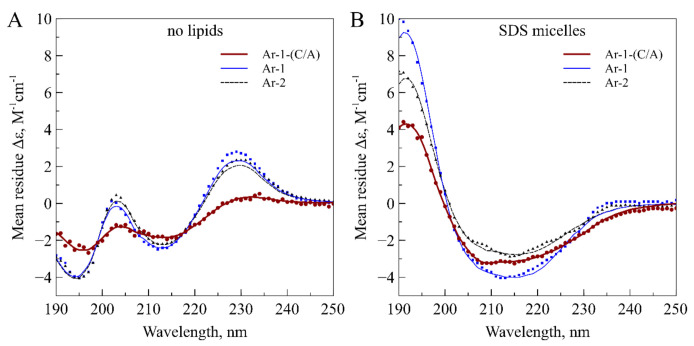
Circular dichroism spectra of Ar-1-(C/A) variant, Ar-1 and Ar-2 in (**A**) aqueous solution and in (**B**) complex with anionic SDS micelles (P:D = 1:350). Experimental data points are shown with symbols and fitted curves calculated with *BeStSel* are pictured as lines. Data for Ar-1 are taken from Panteleev et al. [23] and data for Ar-2 are from Ovchinnikova et al. [24].

We studied the spatial structure of Ar-1-(C/A) and its conformational transitions upon membrane binding by circular dichroism spectroscopy in aqueous solution and in a lipid environment modeled by anionic SDS micelles. To allow the direct comparison with homologs, we analyzed experimental CD data previously collected for wild-type Ar-1 [23] and Ar-2 [24] in similar experimental conditions. The CD spectrum of Ar-1-(C/A) in aqueous solution, shown in Figure 2A, is quite uncommon and has two positive bands at approximately 205 and 230 nm and two negative bands at 195 and 215 nm. This spectrum is remarkably similar to that of wild-type Ar-1 and Ar-2, which are highly right-twisted and kinked β-hairpin molecules in aqueous solution [23,24,25,26], yet the amplitude of the CD signal for Ar-1-(C/A) is lower.

For a more detailed structural analysis, we performed spectra deconvolution using the β-structure selection method [27] recently implemented in the BeStSel web server [28]. As seen from Table 1, BeStSel produced a good fit to the experimental data according to normalized root mean square deviations (NRMSDs), and its estimates of the secondary structure components are given in Table 1.

According to this analysis, Ar-1-(C/A) adopts a predominantly antiparallel β-sheet structure with a considerable right twist in aqueous solution. The experimentally observed propensity of Ar-1-(C/A) to form a β-hairpin structure even without the disulfide bond supports the previous molecular dynamics simulations showing the rapid folding of linear Ar-1 with alkylated cysteines into its native conformation [29]. Indeed, the average content of β-sheet structures in Ar-1-(C/A) is lower than in wild-type arenicins, where disulfide bonds restrict the conformational space and confer additional stability to the β-hairpin.

Upon binding to anionic micelles, Ar-1-(C/A) undergoes a conformational transition which partly transforms the CD spectrum (Figure 2B). The spectrum of micelle-bound Ar-1-(C/A) again resembles that of arenicins in complex with anionic SDS or zwitterionic dodecylphosphocholine (DPC) micelles [23,24,25,26]. The deconvolution of the CD spectrum also gives prevalent β-sheets and turns, however some α-helical characteristics are also detected. Although the helical content is relatively low in all three peptides, the stronger dichroism signal from the α-helices leads to significant transformation of CD spectra of micelle-bound peptides.

Another interesting structural feature of the micelle-bound Ar-1-(C/A) is the decrease in right-twisted antiparallel β-sheet content and a simultaneous increase in the proportion of relaxed antiparallel and parallel β-sheets. We anticipate that these changes in the distribution of various β-sheet types evidence the formation of a more planar structure, which was previously observed for membrane-bound Ar-2 dimers by NMR [25].

**Figure 3:**
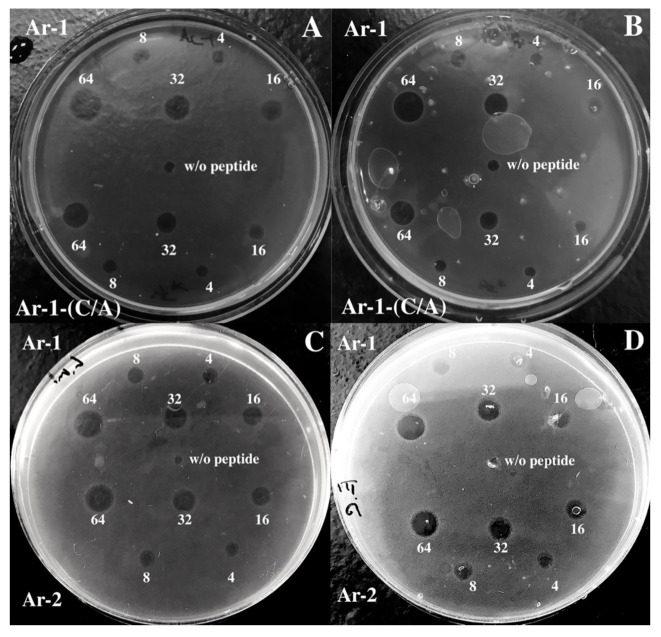
Results of radial diffusion antimicrobial assay against (**A**,**C**) *L. monocytogenes* EGD, (**B**,**D**) *E. coli* ML-35p. Ar-1, Ar-2 and Ar-1-(C/A) were added at concentrations 64, 32, 16, 8 and 4 μM. As a negative control (w/o peptide), deionized water was used.

We measured antimicrobial activity for arenicins using the radial diffusion assay (Figure 3) for measuring the minimum inhibitory concentration (MIC). This method is not very canonical but it provides the data about the antimicrobial activity in solid media. According to the results presented in Table 2, the MICs of Ar-1, Ar-2 and Ar-1-(C/A) against *Escherihia coli* ML-35p are 3.3 ± 1.3, 1.7 ± 0.3 and 4.0 ± 0.5 μM, respectively. The MICs of Ar-1, Ar-2 and Ar-1-(C/A) against *Listeria monocytogenes* EGD are 2.2 ± 0.8, 2.0 ± 0.01 and 3.6 ± 0.8 μM, respectively (see Table 2). Therefore, Ar-1-(C/A) has a slightly but insignificantly weaker activity against both Gram-negative and Gram-positive bacteria than the natural peptides.

The studied peptides possess different hemolytic activity against human erythrocytes (Table 2). The two naturally occurring peptides, Ar-1 and Ar-2, appeared to be more hemolytic (MHC 2.9 ± 1.2 and 1.8 ± 0.9 μM, respectively) than the modification lacking the disulfide bond, Ar-1-(C/A) (MHC 11.1 ± 2.3 μM).

**Figure 4:**
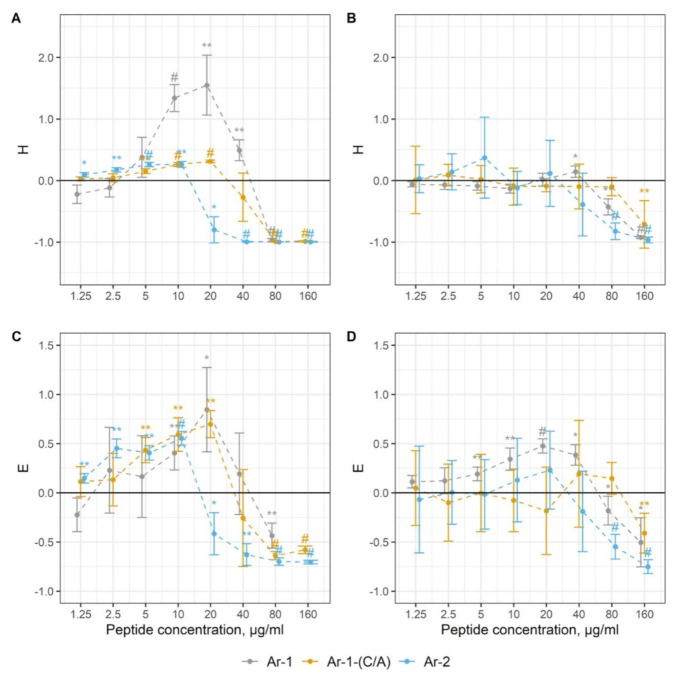
The action of arenicins on complement activation, expressed in H and E coefficients. Data are represented as mean ± standard deviation (*n* = 5). * *p* < 0.05; ** < 0.01; # *p* < 0.001 (H- and E-values vs. zero). (**A**) Alterations in lysis of antibody-sensitized sheep erythrocytes (E^sh^) (CP model); (**B**) alterations in lysis of rabbit erythrocytes (E^rab^) (AP model); (**C**) C3a accumulation in the model with E^sh^; (**D**) C3a accumulation in the model with E^rab^.

In the assay with antibody-sensitized sheep erythrocytes (Er^sh^), which was the model of the classical pathway (CP) activation, the dose-dependent modulation of the complement-mediated hemolysis by the peptides was observed. In Figure 4A, we see in the graph that if the line is above zero, there is complement activation and when it is below zero, that means inhibition of the hemolysis in this test system. As we can see from Figure 4A, Ar-1 led to a significant augmentation of the hemolysis at lower concentrations, but almost abolished Er^sh^ lysis at higher concentrations. Almost the same picture was observed for Ar-2 and Ar-1-(C/A) but the concentrations were slightly different from Ar-1.

To confirm the contribution of complement to the hemolysis, we utilized an ELISA system for human anaphylatoxin C3a detection. In Figure 4C, there is a graph that is similar to that in Figure 4A: a line above zero means active C3a accumulation and complement activation and a line below zero means complement inhibition. Thus, from Figure 4C, we can easily see that in the Er^sh^ hemolytic assay, the elevation of C3a production was observed in the presence of Ar-1, Ar-2 and Ar-1-(C/A) at relatively low concentrations. It was diminished by all arenicin peptides at concentrations corresponding to those at which they abolished hemolysis.

The hemolytic assay with rabbit erythrocytes (Er^rab^) was used as the model of the alternative pathway (AP) activation. It was similar to that for the Er^sh^ lysis assessment and the graph in Figure 4B has the same features. As we can see in Figure 4B, all arenicins demonstrated a strong inhibitory activity at high concentrations. However, it was also shown for Ar-1 that it could slightly activate the complement at a lower concentration. This effect was not detected for Ar-2 and Ar-1-(C/A).

In the Er^rab^ hemolytic assay, ELISA revealed a slight C3a elevation in the presence of Ar-1 at lower concentrations but this was not observed for Ar-2 and Ar-1-(C/A). The C3a level was significantly reduced by the peptides at the concentrations at which they diminished Er^rab^ lysis (Figure 4D).

Importantly, none of the peptides themselves led to hemolysis in experimental models since the lysis level did not differ from the baseline when active serum was replaced by heat-inactivated serum and none of the peptides per se generated a signal in the ELISA system.

**Figure 5:**
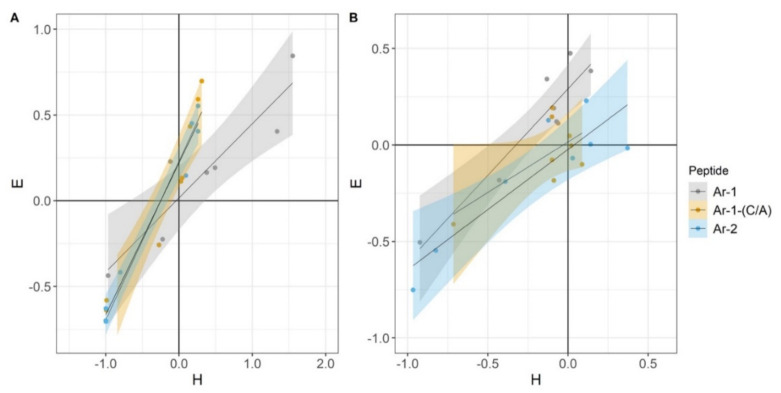
Correlation between H- and E-indexes. These coefficients were used for evaluation of the hemolytic activity of complement (H) and of complement-dependent C3a accumulation (E). (**A**) Correlation between the indexes in the E^sh^ CP model. Pearson correlation coefficient values were calculated as 0.92 for Ar-1, 0.96 for Ar-1-(C/A) and 0.99 for Ar-2. (**B**) Correlation between the indexes in the E^rab^ AP model. Pearson correlation coefficient values were calculated as 0.93 for Ar-1, 0.68 for Ar-1-(C/A) and 0.89 for Ar-2.

In experiments modeling complement activation either via CP or via AP, we observed a good correlation between the level of erythrocyte lysis and C3a accumulation (Figure 5).

Thus, all three peptides demonstrated the ability to modulate complement activation via both pathways. For more details, see Table 2.

## 3. Discussion

One of the main difficulties in using AMPs as therapeutic agents is that they often display high cytotoxic activity towards the host cells and especially erythrocytes. It is possible to lower this effect by modifying the primary structure of AMPs.

We created the arenicin derivative Ar-1-(C/A) to check whether the cysteine bond is critical for (1) the structural stability, (2) biological activity, i.e., antimicrobial function, (3) cytotoxic activity (hemolysis) and (4) the ability to regulate the human complement system. Despite the absence of the disulfide bond, the peptide Ar-1-(C/A) retains the β-hairpin structure, as was shown using CD spectrometry: the spectrum is remarkably similar to that of wild-type Ar-1 and Ar-2 [23,24,25]. However, the average content of β-strand structures in Ar-1-(C/A) is lower than in natural arenicins.

The antimicrobial activity of Ar-1-(C/A) also remained at the level of natural analogs, but the hemolytic activity became 3.5 times lower according to our results. A similar effect was observed earlier by Panteleev and colleagues though they had performed a different modification [30]. It is known that natural Ar-1 and Ar-2 peptides form dimers under membrane-mimicking conditions that usually lead to high cytotoxicity [26]. They created the arenicin derivative Ar-1[V8R] that was less predisposed to form dimers. It was shown to be less cytotoxic for erythrocytes but not for bacterial cells [30].

Another approach was proposed by Lee and co-authors [31]. They demonstrated that one of the ways that could lead to lower cytotoxicity is to replace in the Ar-1 structure Cys3 and Cys20 that form the intramolecular disulfide bond. This modification of Ar-1 without a disulfide bond demonstrated lower antimicrobial and hemolytic activity compared to the natural peptide [31].

Thus, it should be considered that a lower hemolytic activity may correlate with a lower antimicrobial activity due to reduced membranolytic capacity. In this work, we also created an arenicin without a disulfide bond but it appeared that it is not really linear: it is able to form a twisted β-hairpin structure despite the absence of a disulfide bond and it saved the main features that are typical for natural arenicins, except cytotoxic activity against erythrocytes. We anticipate that the lower cytotoxicity is due to the lower stability of the β-hairpin structure in Ar-1-(C/A).

The derivative Ar-1-(C/A) also was shown to retain the ability to modulate the complement system.

In our experiments, we previously observed opposite effects of Ar-1 on complement activation, expressed in the up-regulated or down-regulated hemolysis level and/or up-regulated or down-regulated C3a level that depended on the tested concentration [18]. Here, we found that a similar mode of action is shared by other structurally related peptides, Ar-2 and Ar-1-(C/A).

Natural Ar-1 and Ar-2 differ in a single amino acid residue and both adopt a right-twisted β-hairpin conformation. Nevertheless, a comparison of 3D structures of Ar-1 [32] and Ar-2 [24] determined by NMR suggests that some inequality may exist. The overall conformation of Ar-1 can be described as more compact compared with the more elongated Ar-2 conformation [32]. It remains to be established whether this subtle difference can explain why the effects of Ar-1 and Ar-2 on complement activity are not completely identical.

In our previous works, we showed the ability of Ar-1 to bind C1q [17] and demonstrated its strong inhibitory effect in hemolytic assays for the CP [18]. This fact could serve as evidence that arenicins can lead, through the interaction with C1q, to complement-dependent lysis inhibition and low C3a production. We also found that high doses of Ar-1 inhibit classical and alternative pathways, and the latter cannot be explained only by the interaction of arenicins with C1q. This effect must be related to the action on the common point of both pathways, i.e., C3 cleavage. It is highly probable that arenicins bind the C3 component that leads to its protection from cleavage, although interactions of arenicins with C3 convertases are also possible. Using surface plasmon resonance, we showed that Ar-1 is able to bind C3b protein [33]. Thus, we can suggest that arenicins can bind C1q and C3 or/and C3b and, in some cases, this can lead to complement inhibition.

**Figure 6:**
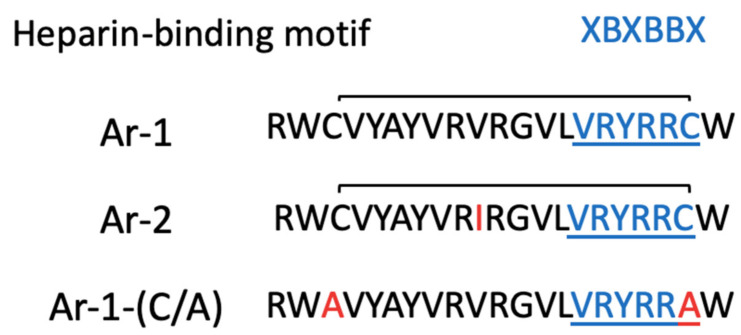
The primary structure of arenicins. Residues in red differ Ar-2 and Ar-1-(C/A) from Ar-1. Underlined residues in blue form a heparin-binding motif.

The ability of arenicins to interact with different complement proteins could be adopted to explain their opposite effects on complement activation. However, there is one more presumable mechanism for complement activation that does not imply direct interaction of the peptides with complement proteins. This effect may be connected with the electrostatic interaction due to which a potent complement inhibitor, heparin (or maybe other GAGs), can be neutralized by peptides that contain the heparin-binding motif XBBXBX, where X—hydrophobic residue, B—basic residue [34]. Indeed, all arenicins contain such motifs, as we can see from Figure 6. A similar mechanism was described for the heparin-binding polypeptide PF4 (platelet factor 4). Although heparin itself is a complement inhibitor [35,36], at the same time, heparin in complex with PF4 was described as activating the complement cascade [37].

The approach involving the use of AMPs as new complement modulators has several advantages. Firstly, these molecules are relatively small, which means that the immune response to these substances will be limited. Secondly, these peptides do not resemble human antimicrobial peptides, which means that these substances would not demonstrate any cross-reactivity and we can avoid a number of side effects. Thirdly, this approach will also help to provide microbicidal or at least bacteriostatic conditions, considering the inhibited complement system. This point is extremely important because using complement inhibitors usually leads to recurrent bacterial infections [38].

## 4. Materials and Methods

### 4.1. Arenicin Peptides

The primary structures of Ar-1, Ar-2 and Ar-1-(C/A) are demonstrated in Figure 6.

The Ar-1 peptide was synthesized using solid phase based on the 9-fluorenylmethoxycarbonyl (Fmoc) protocol with O-(benzotriazol-1-yl)-N,N,N′,N′-tetramethyluronium tetrafluoroborate/N,N diisopropylethylamine (TBTU/DIEA) activation, using Wang resin as the solid phase and triphenylmethyl-protecting groups for cysteines, as described previously [24].

The Ar-2 recombinant peptide was expressed in *Escherichia coli* and then purified as described earlier [24].

The Ar-1-(C/A) peptide was assembled utilizing SymphonyX UV/IR peptide synthesizer (Protein Technologies Inc., Tucson, AZ, USA) running the Fmoc SPPS protocol at a 0.1 mmol scale on Fmoc-Rink amide resin. Side-chain functionalities were protected with tert-butyl (Tyr) groups for tyrosine and a 2,2,4,6,7-pentamethyldihydrobenzofuran-5-sulfonyl group for arginine. Fivefold excess of Fmoc-L-amino acids (Oxyma Pure and DIC), purchased from Iris Biotech (Marktredwitz, Germany), was used for the coupling steps, with NMP as a solvent. After chain assembly, the full deprotection and cleavage were carried out with TFA/H2O/thioanisole/TIS (92.5:2.5:2.5:2.5 *v*/*v*, 120 min, rt). Initial steps of peptide isolation were performed according to the standard procedure in Fmoc chemistry: precipitation with cold diethyl ether, dissolution of the peptide in 0.1 M acetic acid and lyophilization.

Analytical reversed-phase HPLC was performed on C18 columns (4.6 × 100 mm, 3.5 µm, Waters, Milford, MA, USA) in a System Gold 125/166 (Beckman coulter, Pasadena, CA, USA). As solvent A, 0.1% TFA in water was used and as solvent B, 0.1% TFA in acetonitrile. The elution was held using a linear gradient of 5–70% of solvent B to solvent A over 65 min at a 1 mL/min flow rate with UV detection at 235 nm. For the peptide Ar-1-(C/A), identification mass spectrometry was used. Mass spectra were obtained on the maXis impact Q-TOF mass spectrometer (Bruker Daltonics GmbH, Bremen, Germany), equipped with an electrospray ionization (ESI) source (Bruker Daltonics GmbH, Bremen, Germany), operated in positive ionization mode. Mass calibration was carried out with a sodium formate solution (Calibration Mode HPC, standard deviation: 0.336 ppm). Flow injection mode was used for peptide analysis, mass range from 50 to 2000 m/z. Mass spectra were analyzed and created for a neutral molecule using DataAnalysis^®^ software (Bruker Daltonics GmbH, Bremen, Germany).

All peptides were stored lyophilized and were reconstituted using deionized water before use.

### 4.2. Serum and Erythrocytes

Normal human serum used as a source of complement was collected from 30 healthy volunteers, pooled, aliquoted and stored at −70 °C. Erythrocytes were purified from whole blood of rabbit, sheep and healthy donors. The fresh blood was mixed with Alsever’s solution (1:2) and stored at 4 °C for no more than 5 days. Before use, we obtained erythrocytes from the blood and washed them with an appropriate buffer: DGVB^++^ (dextrose gelatin veronal buffer with Ca^2+^ and Mg^2+^: 5 mM sodium barbital buffer containing 150 mM NaCl, 15 mM glucose, 1 mM MgCl_2_, 0.15 mM CaCl_2_, 0.05% gelatin; pH 7.35) for sheep erythrocytes (E^sh^); GVB^+^ (gelatin veronal buffer with Mg^2+^: 5 mM sodium barbital buffer containing 150 mM NaCl, 10 mM Mg-EGTA, 0.05% gelatin; pH 7.35) for rabbit erythrocytes (E^rab^) and PBS (phosphate buffered saline; pH 7.4) for human erythrocytes (E^hum^). Sheep erythrocytes were sensitized with antibodies (anti-sheep red blood cell stroma antibodies produced in rabbits, S1389, Sigma, St. Louis, MO, USA) before use in experiments; we used a 1:1600 dilution of these antibodies and incubated sheep erythrocytes for 30 min at 37 °C.

### 4.3. Circular Dichroism Spectroscopy

The secondary structure of Ar-1-(C/A) was studied by CD spectroscopy. To the estimate conformational transition of the peptide under membrane-mimicking conditions, spectra were obtained either in a buffer alone or one containing anionic SDS micelles. Samples were prepared in 5 mM sodium phosphate buffer (pH 7.5) with 100 mM NaF. Peptide and detergent solutions were mixed, giving final peptide and sodium dodecyl sulfate (SDS) concentrations of 87 μM and 30 mM (P:D ≈ 1:350) and incubated for 1 h at room temperature before the data collection. Circular dichroism spectra were acquired on a Chirascan instrument (Applied Photophysics, Leatherhead, Surrey, UK) in the range 190–250 nm with a 1 nm step size and bandwidth at a 1 s per nm scan rate. The spectra were averaged over five measurements, converted to Δε units and analyzed using the BeStSel web server [24,25].

### 4.4. Antimicrobial Assay

To compare the antimicrobial activity of Ar-1, its modification Ar-1-(C/A) and Ar-2, we used the radial diffusion assay that identifies the antimicrobial activity in the solid medium [39]. For the experiment, the log-phase cultures of *Escherichia coli* ML-35p and *Listeria monocytogenes* EGD were prepared in 3% tryptone soya broth (TSB) at 37 °C for 2.5 h. After this incubation, bacteria were centrifuged at 760× *g* for 12 min at 4 °C to purify them from the medium, then they were washed in 10 mM sodium phosphate buffer (pH 7.4) and centrifuged in the same conditions. After this preparation, bacteria were mixed with warm (43 °C) melted 1% agarose to a final concentration of 4 × 10^5^ CFU/mL. This suspension was poured in Petri dishes and they were left till the agarose became solid. Several wells were cut in this solid agarose layer (4 mm), then peptides were added to these wells at concentrations of 64, 32, 16, 8 and 4 μM. After a 2 h incubation at 37 °C, the mixture of 1% melted agarose and 6% TSB was added onto the Petri dishes to form another layer with nutrients. Three independent experiments were performed.

After the overnight incubation, the diameters of the wells and the zones of growth inhibition were measured. The antimicrobial activity (AMA) was counted as:

AMA = (D − d) × 10

where “D” is the diameter of the zone of inhibition, “d” is the diameter of the well. Minimal inhibitory concentration (MIC) was measured using the dependence of AMA on a peptide concentration, where lg[C_peptide_] is its decimal logarithm. Using a linear regression method, a line was drawn through the points, and the intersection point of this line with the abscissa axis was found. The concentration value for this point was taken as the MIC.

### 4.5. Hemolytic Activity of Peptides

For the evaluation of hemolytic activity, fresh human erythrocytes (E^hum^) were used at a final concentration of 1.25%. They were mixed with the peptides that were diluted consistently in PBS. The final concentrations of each peptide were 100, 25, 5 and 1 μg/mL. These samples were incubated for 30 min at 37 °C. The reaction was stopped by the addition of ice-cold PBS in a ratio of 4:1. Then the samples were centrifuged at 500× *g* for 5 min at room temperature. The optical density of the hemoglobin-containing supernatants was measured at 414 nm (OD_414_). As a negative control, we used the sample with E^hum^ in PBS. As a positive control (100% hemolysis), we took the sample with the E^hum^ but the reaction was stopped by the addition of distilled water instead of PBS.

Hemolytic activity was calculated as:

(OD_414(sample)_ − OD_414(neg.contr.)_) × 100%/(OD_414(pos.contr.)_ − OD_414(neg.contr.)_)

The minimal hemolytic concentration (MHC) was the concentration of the peptide leading to 10% hemolysis. Three independent experiments were performed.

### 4.6. Complement Activation

The ability of peptides to modulate the human complement system was evaluated in hemolytic assay systems and by ELISA, as previously described [18], except that we slightly modified the buffer composition for the hemolytic assay in the model of the classical pathway activation and introduced a novel way for the analysis and visualization of results. For these purposes, we utilized coefficients for the evaluation of the hemolytic activity of complement (H) and of complement-dependent C3a accumulation (E). The hemolytic activity of serum in a sample was measured as

H = (OD_414(sample)_ − OD_414(control)_)/OD_414(control)_

The control was a sample with no peptides added. H values above zero indicate augmentation of complement-mediated hemolysis, while H values below zero mean inhibition. H = −1 corresponds to the complete inhibition of complement-mediated hemolysis and H = 1 corresponds to its two-fold augmentation. The alterations in C3a accumulation were expressed as

E = (OD_450(sample)_ − OD_450(control)_)/OD_450(control)_

As the control, a sample with no peptides added was used. As with the H coefficient, E values above zero indicate an increase in C3a accumulation and E values below zero show a decrease in C3a accumulation. E = −1 corresponds to abolished C3a generation. In practice, reaching this value seems elusive as a small amount of C3a pre-exists in serum due to the spontaneous complement activation. E = 1 corresponds to the two-fold augmentation of C3a accumulation.

### 4.7. Statistical Analysis

Statistical analysis was done using the R language (v4.0.2) in an RStudio environment (R Core Team, R Foundation for Statistical Computing, Vienna, Austria). The significance of H- and E-indexes’ deviation from zero was evaluated by a one-sample *t*-test. The experiments on complement modulation were performed at least five times for each of the peptides. For both hemolytic and ELISA assays, *p*-values less than 0.05 were considered statistically significant. To confirm the link between hemolysis and complement activation, the Pearson correlation coefficient was used. The *p*-values less than 0.05 were considered statistically significant. The plots were drawn using the R language with ggplot2 (v3.3.2) and ggpubr (v.0.4.0) packages.

## Figures and Tables

**Table 1:** The content of secondary structure elements in Ar-1-(C/A), Ar-1 and Ar-2 estimated by the deconvolution of CD spectra with *BeStSel*.

Peptide		α-Helix, %		β-Sheet, %				Turn, %	Other, %	NRMSD
		Regular	Distorted	Antiparallel			Parallel			
				Left-Twisted	Relaxed	Right-Twisted				
Ar-1-(C/A)	aqueous solution	1.5		45.2				9.0	44.2	0.034
		0.0	1.5	1.4	12.2	23.8	7.8			
	SDS micelles	11.8		22.5				11.9	53.7	0.015
		6.5	5.4	0.0	9.1	5.0	8.4			
Ar-1	aqueous solution	0.0		66.0				0.0	34.0	0.034
		0.0	0.0	3.6	24.0	38.4	0.0			
	SDS micelles	15.5		41.7				8.3	34.5	0.018
		15.5	0.0	4.5	22.9	14.3	0.0			
Ar-2	aqueous solution	0.0		67.4				0.0	32.6	0.027
		0.0	0.0	3.9	24.6	38.9	0.0			
	SDS micelles	7.8		44.8				11.7	35.7	0.016
		7.5	0.4	5.0	22.9	16.8	0.0			

**Table 2:** Biological action of Ar-1, Ar-2 and Ar-1-(C/A). ↑—activation; ↓—inhibition; MHC—minimal hemolytic concentration; MIC—minimal inhibitory concentration. All the concentrations are in μM.

Peptide	Modulation of Human Complement System				MHC	MIC	
	Classical Pathway		Alternative Pathway			*E. coli*	*L. monocytogenes*
	Hemolysis	C3a	Hemolysis	C3a			
Ar-1	↑3.6—14.5 ↓29	↑3.6—7.2 ↓29	↑14.5 ↓29–58	↑1.8—14.5 ↓29—58	2.9 ± 1.2	3.3	2.2
Ar-2	↑0.5—3.6 ↓7.2—57.7	↑0.5—3.6 ↓7.2—57.7	↑no ↓28.8—57.7	↑no ↓28.8—57.7	1.8 ± 0.9	1.7	2.0
Ar-1-(C/A)	↑1.9—7.4 ↓29.7—59.3	↑1.9—7.4 ↓29.7—59.3	↑no ↓59.3	↑no ↓59.3	11.1 ± 2.3	4.0	3.6
